# Getting closer: compassion training increases feelings of closeness toward a disliked person

**DOI:** 10.1038/s41598-023-45363-1

**Published:** 2023-10-26

**Authors:** Patricia Cernadas Curotto, Eran Halperin, David Sander, Olga Klimecki

**Affiliations:** 1 Swiss Center for Affective Sciences, University of Geneva, Chemin des Mines 9, 1202 Geneva, Switzerland; 2 Laboratory for the Study of Emotion Elicitation and Expression, Department of Psychology, University of Geneva, Boulevard du Pont d’Arve 40, 1205 Geneva, Switzerland; 3 Psychology of Intergroup Conflict and Reconciliation Laboratory, Department of Psychology, The Hebrew University of Jerusalem, Jerusalem, Israel; 4 Department of Developmental Psychology, Institute of Psychology, Friedrich Schiller Universität Jena, Am Steiger 3, Haus 1, 07743 Jena, Germany

**Keywords:** Psychology, Human behaviour

## Abstract

Evidence-based interventions to favor more harmonious interactions in difficult relationships remain scarce. This study examined whether compassion training may have beneficial effects in an ongoing tense relationship with a disliked person, by reducing schadenfreude toward them and increasing felt interpersonal closeness. 108 participants were assigned to one of three 5-week trainings in a longitudinal randomized controlled study: compassion training, reappraisal training (emotion regulation control condition), or Italian language training (neutral active control condition). The disliked person was not targeted during the trainings to test potential transfer effects. Misfortune scenarios and a measure of interpersonal closeness were used to test whether schadenfreude and closeness feelings toward a disliked person changed from pre- to post-training, across different experimental and control groups. Only compassion and reappraisal trainees reported a decrease of schadenfreude feelings toward the disliked person compared to their pre-training ratings, no changes were observed in the Italian language training. Importantly, feelings of closeness toward the disliked person increased in the compassion training group compared to the other two groups. This increase of closeness feelings could be a central mechanism for improving social interactions. These transfer effects open new perspectives concerning emotion regulation interventions in conflict resolution.

## Introduction

Social relationships are crucial for humans. So far, studies investigating ways to enhance relationships have mainly focused on romantic couples^1–4^ or on intergroup conflict^5,6^. Ways to improve tense interpersonal relationships with people who are not romantic partners are rarely explored, even though they are prevalent.

To date, theoretical as well as empirical work increasingly emphasizes that emotions are central to all meaningful interactions as they can influence behaviors and thus social relationships^7–9^. Extensive research supports that emotions serve at least two major social functions; They foster social closeness (“affiliation function”) or increase social distance (“social distancing function”) between individuals^7,8^. Emotions that serve to distance are associated with long-term attacks and lead to destructive effects on social relationships^10^. In this context, schadenfreude, defined as the pleasure felt when individuals see the suffering of others^11^, has been considered to facilitate the harming of others and thus the deterioration of social relationships^12,13^. On the other hand, emotions such as love that serve the affiliation function have been linked to more close relationships, more cooperation, and generosity^8,14^.

In light of the relevant implications of emotions for social interactions, emotion-based interventions have been developed to promote better relationships. Previous studies tested the effects of emotion regulation strategies, such as cognitive reappraisal training (i.e., reinterpreting a situation that triggered an emotion in order to modify the emotional impact) in conflicts (for instance in couple conflicts, see Ben-Naim et al.^15^). Studies indicate that cognitive reappraisal is a beneficial training to enhance the quality of social interactions and to promote closer social relationships^16^. Indeed, adopting reappraisal techniques has been shown to reduce trait vengeance^17^, to perceive criticism with a more constructive perspective^18^, and to preserve the quality of the relationship over time^4^.

In line with this body of research, a theoretical review has pointed out another emotion regulation strategy^19^, namely compassion training, as a new potential avenue to attenuate tense relationships^20^. Indeed, although there are different conceptualizations of compassion^21^, researchers agree that compassion and its training involve a relational aspect at its core^22^. Here, we define compassion as the feeling of concern for other’s suffering accompanied by a motivation to help^23^. Similarly, self-compassion is defined as the feeling of concern for one’s own suffering coupled with a motivation to help oneself^24^. Both compassion and self-compassion traits have been associated to a series of benefits for social interactions: reduced punishment behaviors^25^, enhanced social connectedness^24^, and a greater tendency to resolve conflicts through compromise^26^.

Consistent with this idea, cultivating compassion has been suggested to benefit social relationships^27^. At the empirical level, several studies support this positive impact of compassion training on social relationships, finding a causal link between compassion interventions (through loving-kindness and compassion-based meditation) and prosocial behaviors^28,29^. Furthermore, longitudinal studies in which compassion has been cultivated have shown that compassion training promotes interpersonal closeness^27,30,31^, in line with the “affiliative function” of emotions. However, even though research on compassion has significantly increased in the last decades^32^, there is still uncertainty whether compassion training also benefits social ties in more difficult contexts such as tense relationships with disliked persons.

Promising evidence comes from a study, which is, to the best of our knowledge, the only study that tested the effect of compassion meditation toward a negative target, a transgressor^33^. In this study, female participants who took part in a compassion training expressed significantly more positive emotions in letters toward a transgressor compared to individuals in other control conditions. Although lacking a pre-post comparison, Koopmann-Holm et al.^33^ provided preliminary evidence that the effects of compassion training can extend to negative targets such as a transgressor. In addition, a study conducted at the intergroup level showed that loving-kindness meditation reduced implicit biases of White US Americans toward Black people even though Black people had not been targeted in the training^34^. This transfer effect suggests that compassion training may be particularly interesting for improving social ties because it does not require explicitly addressing the negative target. This latter point could avoid that efforts for direct conflict resolution backfire^35^.

Therefore, it would be important to test whether compassion training can also have beneficial transfer effects in an actual, ongoing, and tense relationship with a disliked person. In other words: can compassion training change emotions and attitudes when they are directed to a disliked person? Among the emotions that arise from dislike, researchers have identified schadenfreude^36^ which itself is considered a destructive emotion for social interactions^12^. To date, few studies have attempted to reduce schadenfreude feelings (for exceptions, see Greitemeyer et al.^37^, van Dijk and van Koningsbruggen et al.^38^). Nevertheless, several avenues to decrease schadenfreude have been suggested, including interventions aimed at facilitating contact and perceived similarity^39^. Based on the evidence that compassion training benefits social ties by promoting interpersonal closeness^30,31^, compassion training might downregulate schadenfreude and enhance feelings of closeness, even in tense situations, such as with a disliked person.

### The present study

Consequently, the purpose of this randomized controlled trial (RCT) study was to test the efficacy of a compassion training (intending to cultivate benevolent wishes toward a benefactor, oneself, and all living beings) in a difficult interpersonal context such as the relationship with a disliked person. To improve the ecological validity of our research, participants were instructed to select a person they disliked, i.e., a person with whom they had personally interacted and with whom they may have had a conflict (as opposed to selecting an imagined or unknown disliked person). We aimed to determine whether compassion training changed schadenfreude and feelings of closeness toward this disliked person even if the disliked person was not targeted during the training (transfer effect). Schadenfreude feelings were measured using misfortune scenarios involving the disliked person, a typical paradigm in schadenfreude research. Closeness feelings were assessed with the Inclusion of the Other in the Self Scale^40^, a measure that has proven to be a highly reliable measure of subjective closeness in social relationships^41^, and used in prior work testing compassion training effect on social connectedness^31^.

The current study tested the efficacy of compassion training against a reappraisal training and a neutral active control group (i.e., learning Italian) to overcome one of the main limitations in meditation research, which is the lack of RCT with an active control group^33,42,43^. The reappraisal training was included in the study to compare compassion training effects with an emotion regulation intervention whose positive effects have been widely investigated in the context of social interactions^4,16,18^. The neutral active control group (i.e., learning Italian) was included to match the two emotion regulation interventions (compassion training and reappraisal training) on basic nonspecific factors (such as training dosage or group dynamics), except for the emotion regulation component. This allowed for better discrimination between the effects of emotion regulation interventions on the measures of interest and a neutral intervention.

Our hypotheses were that both compassion training and reappraisal training compared with an active control training (i.e., learning a non-native language), would decrease schadenfreude feelings toward a disliked person. In addition, we hypothesized that both compassion and reappraisal training compared to the control condition would lead to an increase in interpersonal closeness toward the disliked person. Finally, because this is the first study of its kind to compare compassion training and reappraisal training in tense ongoing relationships, we explored the differences between the two emotion regulation interventions without a directed hypothesis.

## Method

### Participants

**Figure 1:**
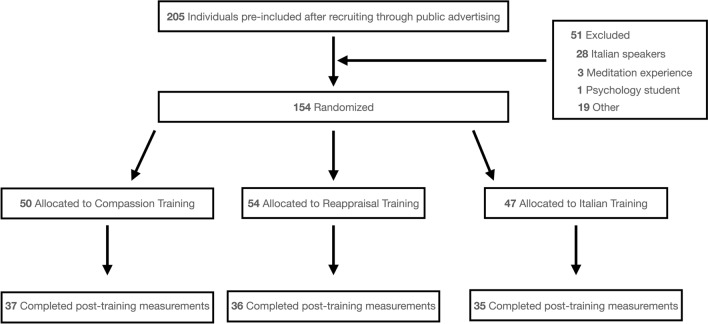
Participants with previous meditation experience, psychology students, and Italian speakers were excluded. “Other” category includes reasons such as some individuals participated in a similar study and knew the study aims or some participants withdrew at this stage.

Volunteers were recruited through advertisements in Geneva as well as in surrounding areas. Participants with previous meditation experience, psychology students, and Italian speakers were excluded. A power analysis was conducted with G*Power, based on an effect size (0.76) reported for a comparison between a control group (waitlist) and a reappraisal training group in a study with similar measures and design^5^ (here, the effect size used was referring to the differences between the groups on negative emotions toward an outgroup member). This analysis suggested that a minimal sample size of 29 participants per group would be required for detecting a standardized difference of negative emotions toward others at significance levels of 0.05. In order to account for potential dropouts and to provide sufficient power for additional outcomes of interest, we decided to include more participants than required by the power analysis. We recruited a total of 205 individuals who were interested in participating in the study. Among them, participants with previous meditation experience, psychology students, and Italian speakers were excluded (*n* = 51). 154 participants were then randomly allocated to one of the three conditions: compassion training (*n* = 50), reappraisal training (n = 54), or Italian training (*n* = 47). Randomization into the groups was performed by using a computer-generated list of random numbers. This information is summarized in a consort flow, see Fig. 1. The study had a dropout rate of 29.87% over the 5-week period of training (more precisely, 26% for the compassion training, 33.33% for the reappraisal training, and 25.53% for the active control training).

At the end, a total of 108 participants (60 females, 48 males; *mean age* = 24.13 years, *SD* = 5.66 years) completed the present study. Participants were randomly assigned to one of three groups: compassion training (23 females, 14 males; *mean age* = 23.57 years, *SD* = 4.79 years), reappraisal training (20 females, 16 males; *mean age* = 22.67 years, *SD* = 4.07 years), or Italian training as an active control training (17 females, 18 males, *mean age* = 26.23 years, *SD* = 7.22 years). A chi-square test revealed that gender assignment was not different across conditions,* p* = 0.51. The present study was approved by the Ethic Committee of the University of Geneva in October 2017. Each participant signed informed consent forms and was paid 55 CHF (~ 55.5 $). All hypotheses and goals of the study were revealed to participants at the end of the experiment.

### Trainings

The three trainings were delivered by experienced instructors and followed the same structure: they started with an introductory session of 1 h that was followed by two group sessions of 2.5 h of duration each. The three sessions took place over a 5-week period and were complemented with 20 min of guided audio recordings for daily listening during the training. All interventions were equal in time and procedure and took place in rooms of the University of Geneva in order to maximize the similarity between them. Furthermore, the instructors were not involved in the design, analysis, or writing of the study. To control for training adherence, participants were asked to log into a platform daily to specify how many minutes they listened to the audio recordings, as well as to indicate the amount of informal practices during the intervention period (i.e., application of the learned techniques in everyday life). Researchers encouraged participants to engage in daily practice of their respective trainings routines to maintain a consistent practice (see Supplementary Material for more details related to data on the training adherence for each condition).

#### Compassion training

Compassion training consisted of a well-established procedure based on meditation sessions and guided audio instructions led by a compassion-based instructor having over 20 years of experience in teaching meditation^44–46^. During the training sessions, compassion trainees were invited to visualize different targets including a benefactor, oneself, and all living beings. Then, they were asked to cultivate feelings of care, benevolence, and kindness toward these targets. In addition, participants were asked to pay attention to body sensations. Compassion sessions in the group were complemented by audio recordings aiming to promote a daily compassion meditation practice. In addition to the audio-recordings, the instructor invited compassion trainees to integrate informal compassion practices in their daily lives such as sending silently benevolent wishes toward people while queuing in the supermarket.

#### Reappraisal training

The reappraisal training sessions were led by a psychologist and researcher in affective sciences with 2 years of experience in teaching courses on emotion regulation. The reappraisal training was designed based on previous studies using reappraisal interventions^4,6,46^. During the training sessions, reappraisal trainees learned to use reappraisal techniques (e.g., thinking about positive outcomes of an unpleasant event). Then, they were invited to practice each one in order to regulate negative emotions elicited by pictures from the International Affective Picture System (IAPS; Lang et al.^47^) and by video clips from the film library developed by Samson et al.^48^. In addition to that, participants used audio recordings for a daily practice of reappraisal techniques. These audio recordings included guidelines to, first, analyze emotions and thoughts related to a recalled unpleasant event and, second, to reinterpret the event in order to decrease potential negative emotions. To foster informal practice, the instructor provided concrete examples to help and motivate participants to implement reappraisal techniques in their daily life, such as reinterpreting bad weather.

#### Italian training

The content of the control condition was selected based on another study that compared meditation training to foreign language training as an active control group^49^. In the present study, the training sessions were Italian introductory lessons led by an Italian instructor with more than 20 years of teaching experience. During the training sessions, participants practiced basic Italian sentences in order to be able to handle a conversation in Italian such as introducing themselves or ordering food in a restaurant. Similar to the other two trainings, participants were required to practice Italian at home with the help of audio recordings. These recordings comprised different types of exercises such as listening to Italian conversations and learning the correct pronunciation by repeating the sentences they heard. Moreover, the instructor suggested to exercise Italian in daily life, for instance by paying attention to the lyrics of an Italian song*.* See Supplementary Material for further details on the three trainings.

### Measures

#### Schadenfreude feelings

First, participants were asked to identify a familiar disliked person, namely someone with whom they may have experienced a conflict in person and a neutral person, namely someone they barely know (see full description of the instructions in Supplementary Material). Then, participants rated schadenfreude feelings on a scale from 0 (*does not fit me at all*) to 100 (*fits with me perfectly*) after having read misfortune scenarios involving either the disliked or the neutral person. A total of eight misfortune scenarios were presented in a random order during the study. They were constructed based on previous schadenfreude studies and adapted to the Swiss culture^37,50–52^. For example, one misfortune scenario described failure on a very important exam (see Supplementary Material for a full description of the misfortune scenarios used in this study). In order to measure schadenfreude, five statements (e.g., “I enjoy what happened to [disliked (neutral) person name]”) traditionally used in schadenfreude research were administrated^51,52^. Analyses revealed that the five statements were reliable with Cronbach’s alpha ranging from ⍺ = 0.95 to ⍺ = 0.96. In addition, compassion feelings were assessed by one item (“I feel compassion toward [disliked (neutral) person name]”) as a manipulation check controlling whether there was an increase of the compassion feelings among compassion trainees. Schadenfreude and compassion feelings were assessed at pre-training and post-training.

#### Closeness feelings

The Inclusion of the Other in the Self Scale (IOSS; Aron et al.^40^) was used at pre- and post-training in order to test how close participants felt to the disliked person, as well as to the neutral person. The IOSS is a pictorial item that consists of seven pairs of circles ranging from nearly touching to a complete overlap. The circles represent the self in relation with the other. Participants have to select the pair of circles which describes best their relationship with the other.

Other measures were included in the design to either check for potential differences (compassion traits, emotion regulation skills, and prosociality) between conditions after the randomization or were used for exploratory purposes on the effects of the two emotion regulation interventions (negative attitudes, prosocial behaviors, and aggression behaviors). Due to their exploratory nature and because no specific hypotheses were formulated for these measures, details on these measures are provided in the Supplementary Material.

### Procedure

Individuals interested in the study received a Qualtrics link to an online demographic questionnaire via email. Participants who met inclusion criteria were then asked to identify a disliked person and a neutral person. Then, participants were invited to a first session in the laboratory for pre-training measures one week before the beginning of the trainings. For each laboratory session, a maximum of 8 participants were invited. Participants assessed their emotions (schadenfreude and compassion) after reading misfortune scenarios involving the disliked person and identical scenarios involving the neutral person. Then, participants evaluated their feelings of closeness toward the disliked person as well as the neutral person. At the end of the laboratory session, each participant was individually informed by the experimenter (orally) about the training they were randomly assigned to. Participants were then enrolled to their respective training (compassion, reappraisal, or Italian training) for 5 weeks. For post-training measures, participants were invited again to the laboratory and were required to not mention the training they had been following to the other participants as the sessions were grouped. They completed the same measures that were used for the pre-training, namely the emotions felt during the misfortune scenarios, and the feelings of closeness. At the end of the experiment, participants were asked to rate their motivation and interest for the training.

### Statistical analysis

Five one-way ANOVAs were used to examine whether the groups differed in their motivation and interest in the assigned training, in training attendance, formal practice, and informal practice. Differences between groups were then controlled and measures of interest (schadenfreude, and closeness) and the manipulation check (compassion) were analyzed with repeated-measures ANCOVAs. Our hypotheses were tested by planned contrasts. All data were analyzed using Statistica version 14.0.0 and the R version 3.5.1 and the packages “psych”, “car”, & “MBESS”.

### Ethics declarations

This research was approved by the ethics committee of Ethic Committee of the University of Geneva in October 2017 (ethical committee approval No. PSE.20170803.40) and performed in accordance with the Declaration of Helsinki. Written informed consent was obtained after the procedures had been fully explained to each participant.

## Results

### Training

To test whether groups differed in their training adherence, two one-way ANOVAs with condition (compassion, reappraisal, Italian training) as a between-subjects factor, and motivation and interest ratings (separately), as dependent variables were conducted. These analyses did not reveal any difference in terms of motivation or interest for the assigned training (all *p*_s_ ≥ 0.31, see Supplementary Material). Moreover, a one-way ANOVA did not reveal any difference between groups for the attendance. Across all groups, participants attended an average of 95.46% of their respective training session time (approximately 5 h and 45 min, for a total of 6 h). Regarding the home practice (audio recording practice and informal practice), post hoc analyses (Tukey HSD) revealed that compassion trainees had higher scores for audio recording practice (*M* = 13.34 min, *SD* = 9.02 min) compared to the Italian group (*M* = 7.31 min, *SD* = 5.53 min, *p* = 0.005) and the reappraisal group (*M* = 8.57 min, *SD* = 8.66 min,* p* = 0.031). No difference was found between reappraisal trainees and Italian trainees in terms of audio recording practice (*p* = 0.78). We decided to control for the differences found in audio recording practice by including audio recording practice as a covariate in the subsequent analyses. Pertaining to informal practices (number of occurrences per day), post hoc analyses showed that while compassion trainees (*M* = 3.15 occurrence/day, *SD* = 2.57) reported significantly more informal practices than Italian trainees (*M* = 1.59 occurrence/day, *SD* = 1.57, *p* = 0.008), there were no other significant differences between groups, all *p*_s_ ≥ 0.13. To ensure that the differences in informal practice did not impact the effects on the dependent variables, informal practice was also included as a covariate in the following analyses.

### Compassion decreased feelings of schadenfreude toward the disliked person

Two 2*2*3 repeated-measures ANCOVAs and planned contrasts were run to analyze the effects of the trainings on compassion feelings (manipulation check) and schadenfreude feelings in response to the misfortune scenarios. These latter analyses comprised a between-subjects factor condition (compassion, reappraisal, Italian training), two covariates (audio recording practice and informal practice), and two within-subjects factors: time (pre-training, post-training) and target person (disliked person, neutral person).

For compassion feelings, the repeated-measures ANCOVA indicated a main effect of the target person, suggesting that participants felt less compassion toward the disliked person than the neutral person, *F*(1,103) = 38.77, *p* < 0.001, *η*_*p*_^2^ = 0.27. No significant main effect of condition, *F*(2,103) = 0.14, *p* = 0.87, *η*_*p*_^2^ = 0.003, nor a significant main effect of time, *F*(1,103) = 0.07, *p* = 0.79, *η*_*p*_^2^ < 0.001, were found. There was no significant effect of the two covariates (audio recording practice, informal practice), *p*_s_ ≥ 0.13. The three-way interaction condition × time × target person and the interaction condition × time were not significant (*F*(2,103) = 0.30, *p* = 0.74, *η*_*p*_^2^ = 0.006, *F*(2,103) = 1.33, *p* = 0.27, *η*_*p*_^2^ = 0.025, respectively). Independent* t*-tests did not indicate any significant difference between groups at baseline, all *p*_s_ ≥ 0.91*.* A planned contrast (post- versus pre-training) for manipulation check showed that compassion trainees were the only participants experiencing a significant pre-post increase of compassion feelings toward the disliked person, (*Mpre* = 32.61, *SDpre* = 24.25; *Mpost* = 41.41, *SDpost* = 26.34, *t*(103) = 2.06 *p* = 0.04, *d* = 0.32). None of the other comparisons or effects were significant, all other *p*_s_ ≥ 0.12.

**Figure 2:**
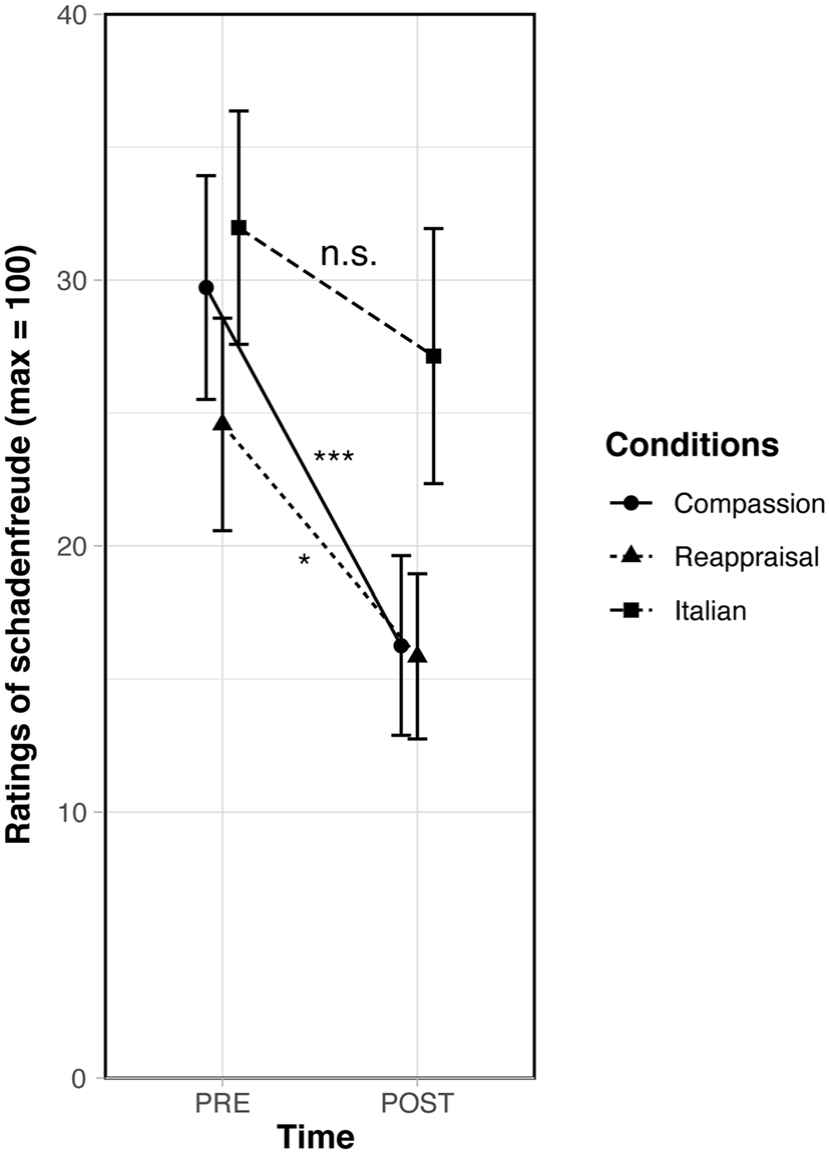
Self-reports of schadenfreude feelings at pre- and post-training toward the disliked person as a function of condition (compassion, reappraisal, Italian). Errors bars indicate ± 1 *SEM.* Asterisks indicate significant changes in time (***p < 0.001, *p < 0.05) and n.s. indicates statistically non-significant differences in time.

We then ran similar analysis with schadenfreude feelings as a dependent variable. The repeated-measures ANCOVA yielded a main effect of target person, *F*(1,103) = 20.52, *p* < 0.001, *η*_*p*_^2^ = 0.17, reflecting that participants felt more schadenfreude feelings toward the disliked person than the neutral person. In addition, there was a main effect of time, *F*(1,103) = 10.67, *p* = 0.001, *η*_*p*_^2^ = 0.09; with schadenfreude feelings decreasing at post-training across conditions. No main effect of condition was found,* F*(2,103) = 2.18, *p* = 0.12, *η*_*p*_^2^ = 0.04, and no significant effect was present for the covariates (audio recording practice, informal practice), *p*_s_ ≥ 0.80. Moreover, there was no significant three-way interaction between time × target person × condition, *F*(2,103) = 0.68, *p* = 0.51, *η*_*p*_^2^ = 0.01 and no significant interaction between time × condition, *F*(2,103) = 2.37, *p* = 0.10, *η*_*p*_^2^ = 0.04. Independent *t*-tests did not show any difference between the groups at baseline, all *p*_s_ ≥ 0.20. As depicted in Fig. 2, planned contrasts revealed that compassion trainees reported lower schadenfreude feelings toward the disliked person after the training than before the training, *t*(103) = − 3.73, *p* < 0.001, *d* = − 0.57. In addition, a significant decrease in schadenfreude feelings was found for reappraisal trainees, *t*(103) =  − 2.08, *p* = 0.04, *d* = − 0.44. No significant change was found for participants in the Italian group, *p* = 0.22. Moreover, a planned contrast revealed a trend for a difference between compassion trainees and Italian trainees on the change in schadenfreude feelings (post-training minus pre-training), *t*(103) =  − 1.81, *p* = 0.07, *d* = − 0.34. No other statistical differences between the conditions emerged. In addition, there was no significant effect of the two covariates (audio recording practice, informal practice) for schadenfreude analyses as well as compassion analyses, *p*_s_ ≥ 0.13*.*

### Compassion training increased closeness toward disliked others

Likewise, the effects of the trainings on closeness felt toward the disliked person and neutral person were also assessed using a repeated-measures ANCOVA (2*2*3) with audio recording practice and informal practice as covariates. The sample size for this analysis was reduced to 107 participants because of one missing data point at post-test. No significant effect of the target person was found in the current analysis, *F*(1,102) = 0.03, *p* = 0.87, *η*_*p*_^2^ < 0.001. Furthermore, no significant effect of condition, *F*(2,102) = 0.82, *p* = 0.44, *η*_*p*_^2^ = 0.02 was found. The three-way interaction condition × time × target person was not significant, *F*(2,102) = 0.63, *p* = 0.53, *η*_*p*_^2^ = 0.01. However, we found a significant two-way interaction time × condition, *F*(2,102) = 7.64, *p* < 0.001, *η*_*p*_^2^ = 0.13. This finding suggests that participants in the compassion training expressed more closeness feelings in post-training ratings than they did before the training, whereas participants in the other conditions (reappraisal, Italian) reported lower feelings of closeness. In addition, a significant main effect of time was found, *F*(1,102) = 8.19, *p* = 0.005, *η*_*p*_^2^ = 0.07, suggesting that participants experienced changes in interpersonal closeness ratings between pre-training and post-training. Regarding the effects of the covariates audio recording practice and informal practice, no significant effect was found, *p*_s_ ≥ 0.10.

**Figure 3:**
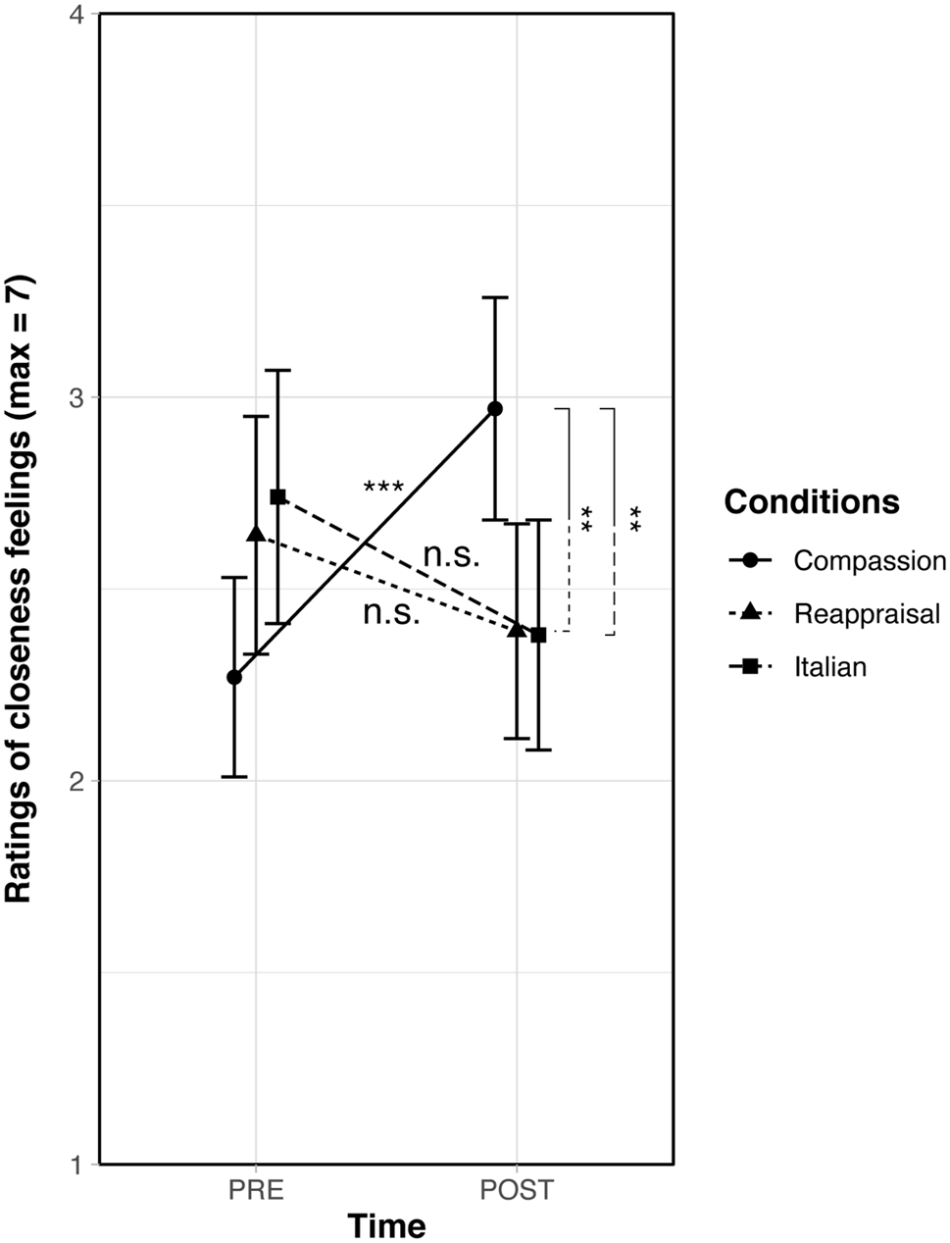
Self-reports of closeness feelings at pre-training and post-training toward the disliked person as a function of condition (compassion, reappraisal, Italian). Errors bars indicate ± 1 *SEM.* Asterisks indicate significant changes in time (**p < 0.01) and n.s. indicates statistically non-significant differences in time.

*T*-tests did not reveal any group difference at baseline, all *p*_s_ ≥ 0.31*.* Planned contrasts (post- versus pre-training) revealed that participants in the compassion training were the only ones to report a significant increase of closeness felt toward the disliked person after the five weeks of intervention, *t*(102) = 2.96, *p* = 0.004, *d* = 0.46 (see Fig. 3). Planned contrasts on the change in closeness feelings (post-training minus pre-training) indicated that compassion trainees increased their feelings of closeness toward the disliked person compared to participants in the reappraisal group,* t*(102) = 2.82, *p* = 0.006*, d* = 0.66, and the Italian group, *t*(102) = 3.03, *p* = 0.003*, d* = 0.75. No significant effect of the two covariates (audio recording practice, informal practice) was found, *p*_s_ ≥ 0.10*.*

## Discussion

The aim of this study was to test whether compassion training can change attitudes and feelings toward a disliked person. Furthermore, we tested here if the beneficial effects of compassion training were transfer effects as the training did not target or mention the disliked person. Our manipulation check confirmed that only compassion trainees expressed an increase of compassion feelings in response to a misfortune of a disliked other after 5 weeks of intervention. In addition, participants in both emotion regulation interventions—compassion and reappraisal trainings—reported less schadenfreude feelings toward this disliked person at post-training compared to their pre-training ratings. Finally, only compassion trainees showed an increase of closeness feelings toward the disliked person at post-training. The findings reported here suggest that compassion training is a beneficial strategy to enhance difficult social relationships and, thanks to its transfer effects, could be considered an indirect conflict resolution intervention.

Here we show for the first time that both emotion regulation interventions (compassion training and reappraisal training) were efficient in decreasing schadenfreude feelings. Future research may study whether the two interventions decrease schadenfreude through distinct mechanisms. Roseman and Steele^39^ proposed that interventions aiming at self-enhancement in a non-competitive way (for an example, see Van Dijk et al.^38^) can reduce schadenfreude. Interestingly, high levels of self-compassion have been proposed to enhance feelings of self-worth^53^. The compassion training used in this study included practices of self-compassion (i.e., moments of cultivation of benevolent wishes toward oneself). Nonetheless, the question remains, did the compassion training used here strengthen self-worth, which in turn reduced schadenfreude? Future work could address this question by including measures of self-compassion and self-worth at pre- and post-training. Regarding how reappraisal training may lead to less schadenfreude, a potential candidate would be a change in perspective. Indeed, one of the techniques taught during the reappraisal training was perspective-taking (i.e., changing the point of view of a situation). For instance, adopting the perspective of the disliked person during the post-training testing might have conducted participants to focus on the negative consequences resulting from the misfortune and consequently, experience a reduction of the pleasure.

Importantly, the increase found in closeness feelings toward the disliked person extends past research in which contemplative interventions were found to promote social connectedness^30,31^. Notably, the increase of closeness feelings found in these previous studies was directed at neutral targets (i.e., other participants or non-specified relationships). The present study, however, went further by revealing augmented closeness feelings for a specific disliked other after compassion training. This finding provides further support for the relational core of compassion training^22^ and suggests that compassion may facilitate the affiliative function of emotions, even in difficult contexts. While disliking people is not inherently bad, it may still be beneficial to mitigate such feelings, as they can lead to adverse consequences for oneself, such as discomfort^54^. One might want to downregulate this unpleasant feeling. Moreover, dislike has also been associated to distancing and hostile responses^55,56^, which, can, in turn, worsen relationships, especially when nonvoluntary interactions are frequent (typically occurring when one must regularly engage with someone they dislike).

Thus, a possible implication of this finding is that increased feelings of closeness may overcome consequences of nonvoluntary interactions and promote constructive dynamics in social relations with disliked others. A next step that future research should address is to use a longitudinal design aimed at testing whether increases in closeness translate into sustained changes in relationships with the disliked person, and result in more constructive interactions.

Conversely, reappraisal participants in the current study did not experience a significant increase in closeness feelings toward the disliked other, differing from previous findings showing that the frequent use of reappraisal techniques leads to closer relationships^16^. A possible explanation for the difference between compassion and reappraisal trainings is the potential of compassion training to increase positive attitudes in response to distressful scenes^28,57^. In contrast, reappraisal training has not only been related to fewer prosocial behaviors^58,59^, but also to less visual attention for suffering situations compared to compassion training^60^. Thus, although reappraisal training may be very beneficial in several contexts^61^, compassion training may be a better strategy to promote adaptative emotions and attitudes in unfortunate situations. Finally, emotion regulation interventions goals might differ on more than one aspect. Indeed, reappraisal training focused mainly on down-regulating negative emotions (only one technique focused on increasing positive emotions) whereas compassion training focused on promoting positive emotions. These differences could explain results on the increase of closeness feelings as positive emotions have been related to promote social inclusiveness^62^. More studies on the specific mechanisms responsible for differential beneficial effects of compassion versus reappraisal trainings in particular contexts would be important for both, fundamental and applied research.

In addition to these potential contributions, the current study also has limitations that could be addressed in future research. This research was limited by the use of self-reports. As schadenfreude may be affected by social desirability due to its attributed immoral nature, scholars have suggested to use implicit measures of schadenfreude such as the use of facial electromyography^13,36,63^. In addition, heart rate variability has been proposed as a useful way to measure compassion training outcome^64^. A further study could assess emotion regulation interventions effects by adopting paradigms using facial electromyography and measures of the autonomic bodily reaction. Importantly, forthcoming research should test the enduring impacts of compassion and reappraisal trainings through longitudinal designs with long-term follow-up employing diverse methods that do not rely solely on self-reported data.

Despite these limitations, the current study shed light on the role of emotion regulation interventions in tense interpersonal relationships. The present findings indicate that reappraisal training and compassion training are efficient interventions to decrease schadenfreude for a disliked person. Additional results suggest that compassion training is a way to promote healthier relationships by enhancing compassion feelings for a disliked person. Furthermore, compassion training acted as “social glue” as it increased closeness feelings toward a disliked person. Importantly, effects of compassion training were found toward the disliked person although the disliked person was not targeted during the training. This generalization is key as it may help to overcome one of the major obstacles faced by peacebuilding scholars and practitioners: the lack of motivation to change attitudes or emotions felt toward foes or outgroup members^65,66^. By extending its effects beyond people targeted during the training, compassion training seems to be a good candidate to counteract the lack of motivation in contexts in which it is difficult to experience constructive social emotions (i.e., empathy). Further work should investigate whether compassion training effects also extend to more difficult and violent situations such as in intergroup conflicts.

### Supplementary Information

Supplementary Information.

## Data Availability

Data used in the present study is publicly available at https://osf.io/cgbvt/.
