# Supplementary material for: Getting closer: compassion training increases feelings of closeness toward a disliked person
Source: Sci Rep. 2023 Oct 26;13:18339. doi: 10.1038/s41598-023-45363-1 (PMC10603062; doi:10.1038/s41598-023-45363-1)
Supplement: Supplementary file 1 — Supplementary Information. [file 41598_2023_45363_MOESM1_ESM.docx]

Getting closer: compassion training increases feelings of closeness toward a disliked person

Patricia Cernadas Curotto, Eran Halperin, David Sander, & Olga Klimecki

Supplementary Material

**Training information**

Compassion Training. In the introductory session, compassion trainees were familiarized with a brief theoretical background on meditation and were then invited to experience a short practice of mindfulness to increase awareness of their bodily feelings. For instance, the instructor encouraged participants to focus on their breath. In the two following trainings sessions, volunteers were engaged in i) visualizing different targets including a benefactor, oneself, and all living beings, and ii) in cultivating feelings of care, benevolence, and kindness toward these targets. More precisely, they were instructed to send wishes such as “May you be safe” or “May you be free of suffering” while they were visualizing these different targets. During these compassion enhancing techniques, participants were asked to pay attention to feelings evoked by these exercises. In addition to in-person sessions, audio recordings were included to promote a daily compassion meditation practice at home. The first audio-recording included two guided compassion practices toward a benefactor and oneself. In the second audio-recording, compassion trainees were invited to engage in techniques aiming to enhance compassion feelings toward a benefactor, oneself, and to extend these feelings to all living beings.

Reappraisal Training. Starting with a brief definition of emotion regulation and reappraisal in the introductory session, the instructor presented 4 reappraisal techniques described by McRae and colleagues (2012) in the following training sessions. The techniques comprised (1) the explicitly positive reappraisal, thinking about the positive outcomes of an unpleasant event, (2) the change of current circumstances, changing one or several aspect of the actual situation, (3) the change of future consequences, reevaluating them as different than one might expected, and finally (4) distancing or perspective-taking, changing the point of view in order to take some distance from the event. Each reappraisal technique was then used to downregulate negative emotions elicited by pictures from the International Affective Picture System (IAPS; Lang, Bradley, & Cuthbert, 2008) in the first training session and by video clips from the film library of Samson and colleagues (2016) in the second training session. Moreover, participants were invited to recall personal unpleasant events while trying to reappraise them with one of the four techniques. Reappraisal trainees used audio recordings for a daily practice of reappraisal techniques at home. In the audio recording, participants were first invited to select an unpleasant event and pay attention to the emotions they felt. Then, they were invited to analyze the thoughts related to a recalled unpleasant event and the audio recording guided them to reinterpret the event to decrease potential negative emotions.

Italian Training. As the importance of including an active control group in studies investigating interventions and in particular meditation-based interventions has been highlighted (Koopmann-Holm et al., 2020; Kreplin et al., 2018), an active control group was added to this procedure (here: Italian training). As participants were not Italian speakers, the training sessions were Italian introductory lessons. In the introductory session, participants learned about the importance of Italian in nowadays, the history of the Italian language, and the Italian culture to increase their motivation to learn Italian. The training sessions focused on an introduction of the grammar and the conjugation in Italian. In addition, participants read, wrote and practiced basic Italian sentences allowing them to introduce themselves, ask for their way, order in a restaurant or handle an easy conversation. To complement in-person sessions, participants were asked to practice Italian at home with the help of audio recordings. The audio-recordings helped participants learn correct pronunciation as they were asked to first listen to and then repeat the sentences to which they had paid attention. Other exercises comprised listening and translation of these sentences to get a better understanding.

Importantly, we ensured that the trainings were as similar as possible: same structure, similar number of participants per training, same rooms. Moreover, we also made sure that the disliked person was not mentioned in any of the trainings.

**Instructions used for identifying the disliked person and the neutral person**

During this experiment we will potentially ask you to think about 2 persons. You will have to estimate your relationship with a person you do not like and a person you do not know well. We would like you to identify them and provide information about the relationship maintained with these people. We remind you that all answers to our questionnaires are confidential.

1. First, think of a person you find difficult to live with. A person with whom you may have had a conflict in the past. Please visualize this person.
   1. Could you please tell us to which social circle (family, workplace, ex-boyfriend/girlfriend, neighbor, other) this person belongs or belonged?
2. Now we're going to ask you to visualize a person you do not know or know very little about. This is a person with whom you have had no experience, either good or bad. An "acquaintance" for whom you do not have a strong feeling, whether good or bad. You have probably met this person in your daily life. This person can be for example your mailman, the cashier of the cafeteria you often frequent, a bus driver, a neighbor you know by sight, a person you pass on the bus or in the street...”

**Misfortune scenarios**

Imagine that two men entered in [target] house and fettered the [target] and forced him/her to communicate where were his/her goods are. Then the two men fled with an important amount of money. (Based on Greitemeyer et al., 2010)

Imagine that [target] had his/her end-of-year exam to obtain his/her diploma for his/her career. [target] went to a party the night before one of the end-of-year examinations and did not revise some key topics for the exam. [Target] did not obtained his/her diploma. (Based on Feather & Sherman, 2002)

Imagine that [target] had his/her end-of-year exam to obtain his/her diploma for his/her career. The exam turns out to be very difficult and [target] finished up with a fail grade and did not obtained his/her diploma. (Based on Feather & Sherman, 2002)

Imagine that [target] has rented an expensive car to “make an entrance” at a student party. After arriving, while trying to park his/her rented car, he/she drove it into the canal. Consequently, the car had to be towed out of the canal by the fire brigade and appeared severely damaged. (Based on van Dijk et al., 2011)

**Supplementary measures**

Participants also completed other measures than the ones presented in the main manuscript. These supplementary measures were either to check for potential differences between the conditions after the randomization (see Baseline Measures) or they were used for exploratory purposes on the effects of the two emotion regulation interventions (see Perceived Threat Scale, Inequality Game, and Dictator Game). No specific hypotheses were formulated for these measures.

Baseline Measures. A series of questionnaires related to personality traits and emotion regulation skills were administrated online before the first visit at the laboratory*.* Compassion traits and fear of compassion were measured with the 7-item Compassion for Strangers Scale (COS-7; Schlosser et al., 2021), and Fear of Compassion Scales (FCS; Gilbert et al., 2011), respectively. The items of the 7-item Compassion for Strangers Scale were rated with a 7-point Likert scale ranging from 1 (not at all true of me) to 7 (very true of me). Pertaining to the Fear of Compassion Scales, the three subscales are: fear of compassion for others (10 items), fear of compassion from others (13 items), and fear of compassion for self (15 items). All the items of the Fear of Compassion Scales were assessed using a 5-point Likert scale from 0 (don’t agree at all) to 4 (completely agree). In order to have a baseline measure of prosocial behaviors, participants responded to the Prosocialness Scale for Adults (PSA; Caprara et al., 2005) composed of 16 items ranging from 1 (never/almost never true) to 4 (almost always/always true). Finally, emotion regulation skills were measured using the Emotion Regulation Questionnaire (ERQ; Gross & John, 2003). The Emotion Regulation Questionnaire comprises two subscales: the reappraisal scale (5 items) and the suppression scale (5 items). Participants indicated on a 7-point Likert scale ranging from 1 (strongly disagree) to 8 (strongly agree) whether they agree with the statements. Scores on these questionnaires are summarized in Supplementary Table 1.

Perceived Threat Scale. To measure the negative attitudes attributed to the disliked person, we used a modified version of the Perceived Threat Scale (PTS; Halperin et al., 2009). Participants gave a name (fictitious or the actual name of the disliked person) to the disliked person. This name was used to adapt the statements. In addition of two items from the original PTS (i.e., “[Disliked person name] proves constantly that he/she is unreliable and is able to turn his/her back at any time”;“[Disliked person name] represents a threat to my integrity”), 5 new items based on hatred literature (Fischer et al., 2018; Halperin, 2016) were included (e.g., “The personality of “[disliked person name] is intrinsically bad”; “If [disliked person name] has made mistake or has harmed, it was intentional”; “I don’t see myself reconcile with [disliked person name]”; “It’s completely his/her fault if there is no compromise that we can do”). Reliability analyses showed that the items of the modified PTS indicated an overall good reliability (⍺ = .72). Participants indicated with a scale from 0 (*not realistic at all*) to 100 (*very realistic*) the extent to which the statements applied to how they perceived the disliked person. This scale was presented twice: at pre- and post-training.

The Dictator Game. Previous studies have investigated prosocial behaviors as outcomes of a compassion training and have shown that compassion training had a beneficial impact on prosocial behavior as measured by the Zurich Prosocial Game and not on norm-based prosocial behavior as measured by the Dictator Game (Leiberg et al., 2011). The Dictator Game (DG; Güth & Huck, 1997) was used in the current study to observe the impact of compassion training on norm-based prosocial behavior. In this version of the DG, participants played the role of the “dictator” and had to divide an imaginary sum of 10 CHF (~10 $) between themselves and the disliked person as well as between themselves and the neutral person. This economic game was played once at post-training.

Inequality Game. The Inequality Game (IG; Klimecki et al. 2016) was played once at post-training and was used to assess four social behaviors in a socio-economic interaction with strangers: cooperation, aggression, punishment, and forgiveness. In the current study, experimenters made participants believe that they were playing the IG with two other individuals present in the same room. In order to reinforce this belief, participants played in a multi-computer room with individuals from different trainings being present (so that participants would not know each other). Participants ignored that the behavior of the “two other players” was preprogrammed to be fair or unfair during the game. In each trial, the participant was paired with one of two another alleged player and a payoff matrix was presented. The two players jointly decided between four possible allocations of amount of money to the players. These allocations were either cooperative (high gain for both players) or competitive (high gain for the player choosing first, low gain for the player choosing second). More precisely, in a first phase of the IG, either the fair or the unfair player was the one making the first economic choice, thus affecting participant’s gain. The fair player always chose cooperative economic outcomes (i.e., high gain for both players) whereas the unfair player always picked competitive economic outcomes (i.e., high gain for self and low gain for the participant). In addition to economic choices, players could also send feedback messages during the game. These latter were either nice (e.g. “You are very nice”) or derogatory (e.g. “You are annoying”). Again, the fair player always picked nice messages and the unfair player derogatory messages. In a second phase of the IG, participants were the first to decide on economic choices. The economic choices made by the participants during this phase could thus be categorized as prosocial, sanctioning, or competitive. Participants who predominantly maximized the gain for both other players were classified as prosocial. Conversely, participants who mainly minimized the outcomes of both other players were categorized as competitive. Finally, participants who selected high gain for the fair player and low gain for the unfair player were categorized as sanctioning.

Prior to the game, experimenters informed participants that two of their economic decisions would be implemented at the end of the game and that this benefit could go up to 20 CHF (~20 $). For fairness considerations, each participant received the same amount of money (~10 $) at the end of the experiment.

**Results**

| **Supplementary Table 1**  *Demographic and Baseline Measures* | | | | | | |
| --- | --- | --- | --- | --- | --- | --- |
|  | **Compassion** | | **Reappraisal** | | **Italian** | |
|  | (n = 37) | | (n = 36) | | (n = 35) | |
|  | *Mean* | *SD* | *Mean* | *SD* | *Mean* | *SD* |
| **Age** | 23.57 | 4.79 | 22.67 | 4.07 | 26.23 | 7.22 |
| **ERQ - Reappraisal** | 5.05 | 1.03 | 4.87 | 1.26 | 4.92 | 1.22 |
| **ERQ - Suppression** | 3.73 | 1.41 | 4.03 | 1.26 | 3.91 | 1.19 |
| **7-item Compassion for Strangers** | 4.38 | 0.90 | 4.37 | 1.13 | 4.46 | 0.95 |
| (*Continued*) | | | | | | |

| **Supplementary Table 1** (Continued) | | | | | | |
| --- | --- | --- | --- | --- | --- | --- |
|  | **Compassion** | | **Reappraisal** | | **Italian** | |
|  | (n = 37) | | (n = 36) | | (n = 35) | |
|  | *Mean* | *SD* | *Mean* | *SD* | *Mean* | *SD* |
| **FC - for self** | 14.46 | 12.40 | 21.25 | 12.75 | 18.22 | 8.62 |
| **FC - from others** | 14.11 | 9.14 | 18.22 | 8.62 | 16.94 | 10.02 |
| **FC - for others** | 20.51 | 9.33 | 20.36 | 6.99 | 21.74 | 8.56 |
| **PSA** | 3.89 | 0.48 | 3.85 | 0.62 | 3.91 | 0.51 |
| *Note*. Means and standard deviations of demographic and baseline measures as a function of condition (compassion, reappraisal, Italian). ERQ = Emotion Regulation Questionnaire; FC = Fear of Compassion; PSA = Prosocialness Scale for Adults; SD = Standard Deviation. | | | | | | |

**Control for potential differences in demographic data and baseline measures**

To ensure that there were no differences between groups regarding demographic data and baseline measures, we conducted one-way ANOVAs as well as Kruskal-Wallis tests when the data was not normally distributed ( i.e., for the age, for the scores of the Fear of Compassion from others, the Fear of Compassion for self, and the Prosocialness Scale for Adults). Means and standard deviations are summarized on Supplementary Table 1. Importantly, four one-way ANOVAs as well as four Kruskal-Wallis tests revealed that groups did not differ on these variables, *p*_s_ ≥ .16, except for their scores on the subscale Fear of Compassion for the self: a main effect of the condition was found, *H*(2) = 6.94, *p* = .03. Therefore, we then calculated pairwise comparisons between groups to observe potential differences using Wilcoxon signed-rank tests (non-parametric analyses) as the data was not normally distributed. These analyses indicated that there was a trend for a difference between reappraisal trainees and compassion trainees on scores of the Fear of Compassion for self, *p* = .051. Thus, we conducted Spearman correlations to observe whether this trend was associated with any of our measures of interest, namely, compassion ratings, schadenfreude ratings, and closeness feelings. Spearman correlations indicated that the scores of Fear of Compassion for self did not correlate with any of our measures of interest, *p*_s_ ≥ .19.

**Training differences between the conditions**

In order to test whether there were differences in our conditions related to training characteristics, a MANOVA with a between-subjects factor condition (compassion, reappraisal, Italian training) and five dependent variables was computed. The dependent variables were the motivation for the training, the interest for the training, the attendance of the session, the audio-recording practice, and the informal practice. As the multivariate analysis using Pillai’s Trace revealed a significant effect for the conditions (*V* = 0.18, *F* (2,105) = 1.97, *p* =.038), one-way ANOVAs for each training measure were performed. Descriptive statistics and group differences are reported in Supplementary Table 2. Post hoc Tukey tests showed that the compassion group differed significantly from the Italian group (*p* = .005) as well as from the reappraisal group (*p* = .031) for the audio recording practice. Regarding to the informal practice, a post hoc Tukey test yielded a significant difference between the compassion group and the Italian group (*p* = .008) while there were no significant differences between compassion trainees and reappraisal trainees (*p* = .13). Consequently, analyses of the measures of interest were conducted with audio recording and informal practice as covariates to control for the differences revealed by post-hoc Tukey tests.

To check for relations between the practice time metrics (formal practice and informal practice) and the key outcome variables (compassion, schadenfreude, and closeness feelings), regressions between these variables were conducted in R. To this purpose differential scores were calculated on main variables (i.e., post-training scores – pre-training scores on compassion, schadenfreude and closeness) and the two practice time metrics (formal practice and informal practice) were treated as moderators. These regressions were ran using the whole sample (N = 108).

The only significant interaction found was the regression model with schadenfreude differential scores. More precisely, the interaction between condition and formal practice was found statistically significant, *F*(2, 102) = 3.97, *p* = .02. To delve deeper into this result, the data was subset based on conditions (Compassion, Reappraisal, and Italian). Pearson analyses were ran on these distinct subsamples, and showed that the only significant correlation was found among Italian trainees (n = 35), *r* = .48, *p* = .004.

| **Supplementary Table 2**  *Training Adherence and Training Practice Measures* | | | | | | | | |
| --- | --- | --- | --- | --- | --- | --- | --- | --- |
|  | **Compassion** | | **Reappraisal** | | **Italian** | | **Group Difference** | |
|  | (n = 37) | | (n = 36) | | (n = 35) | |  |  |
|  | *Mean* | *SD* | *Mean* | *SD* | *Mean* | *SD* | *F* | *p* |
| **Motivation (0 - 100)** | 66.14 | 22.33 | 68.08 | 21.95 | 73.83 | 21.98 | 1.17 | .31 |
| **Interest (0 - 100)** | 73.78 | 24.04 | 73.53 | 22.7 | 78.34 | 23.92 | 0.47 | .63 |
| **Attendance to session (%)** | 95.31 | 10.43 | 96.10 | 11.27 | 94.96 | 9.74 | .11 | .90 |
| **Audio recording (min/day)** | 13.34 | 9.02 | 8.57 | 8.66 | 7.31 | 5.53 | 5.85 | .004** |
| **Informal practice (occurrence/day)** | 3.15 | 2.57 | 2.15 | 2.25 | 1.59 | 1.57 | 4.75 | .011* |

*Note*. Five one-way ANOVAs were carried out to test for differences as a function of condition (compassion, reappraisal, Italian) in training characteristics. Post hoc Tukey Tests showed that compassion trainees practiced significantly more using the audio recordings than Italian trainees, *p* = .005 and that they also reported more informal practices than Italian trainees, *p* = .008.Asterisks indicate significant differences between conditions: ***p* <. 001, **p* <.05

Supplementary analyses

Disliked person characteristics

The disliked person chosen by the participants was most frequently someone from the workplace (25%) or a former friendship (24.07%). Family members were selected as the disliked person in 17.59% cases whereas ex-relationships in 8% of the cases. Neighbors were depicted as the disliked person in 2.77% of the cases. The remaining percentage (23.15%) was not classified in any of the categories. At the end of the experiment a subgroup of our sample (N = 33), responded to an additional item regarding the disliked person: “*Are you still in touch with this disliked person? Please estimate the frequency of your interactions*”. This additional item revealed that one third (n = 11) of the participants continued to regularly see the disliked person (more than once a month).

**No impact of the emotion regulation interventions on perceived threat from the disliked other**

To test the effect of the emotion regulation interventions on perceived threat from the disliked person, a 2*3 repeated-measures ANCOVA was conducted using the mean score of the 7 items of the Perceived Threat Scale (PTS) as the dependent variable. In addition to the between-subject factor condition (compassion, reappraisal, Italian training), a within-subject factor time (pre-, post-training) and two covariates (audio recording practice, informal practice) were included. Results indicated that there was a main effect of the condition, *F*(2,103) = 4.28, *p* = .02, *η_p_*^2^= .08 and a main effect of time, *F*(1,103) = 4.98, *p* = .03, *η_p_*^2^= .05. No effect of the interaction condition × time was found, *F*(2,103) = 0.63, *p* = .53, *η_p_*^2^= .01. *T*-tests revealed a significant difference between the compassion trainees and Italian trainees *(p* = .02) at baseline, indicating that compassion trainees (*Mpre* = 55.37, *SDpre* = 19.84) attributed significantly less threats or hatred traits to the disliked person compared to Italian trainees (*Mpre* = 64.93, *SDpre* = 21.54). All means and standard deviations are reported in Supplementary Table 3. As a consequence, a second ANCOVA was executed: including a between-subjects factor condition (compassion, reappraisal, Italian training), the two covariates (audio recording practice, informal practice), the post-training ratings as the dependent variable, and finally adding the ratings of the PTS at pre-training as a third covariate. The new ANCOVA showed that there was no main effect of the condition when adjusting for the pre-training ratings, *F*(2,102) = 1.71, *p* = .19, *η_p_*^2^= .03. In addition, while there was no significant effect for the covariates related to the training practice (audio recording practice, informal practice), *p*_s_ ≥ .42, the pre-training ratings of the PTS as covariate were significant, *F*(1,102) = 81.24, *p* <.001, *η_p_*^2^ = .44. Planned contrasts with independent *t*-tests revealed a trend for a difference between compassion trainees and Italian trainees, *t*(102) = -1.84, *p* = .069, *d* = -65. Other comparisons between conditions were not significant (all *t*_s_ (102) ≤ -0.80, all *p*_s_ ≥ .26).

| **Supplementary Table 3**  *Perceived Threat From the Disliked Person* | | | | | | |
| --- | --- | --- | --- | --- | --- | --- |
|  | **Compassion** | | **Reappraisal** | | **Italian** | |
|  | (n = 37) | | (n = 36) | | (n = 35) | |
|  | *Mean* | *SD* | *Mean* | *SD* | *Mean* | *SD* |
| **Pre-training** | 55.37 | 19.84 | 57.90 | 17.11 | 64.93 | 21.54 |
| **Post-training** | 46.66 | 18.92 | 52.56 | 20.12 | 60.89 | 24.91 |
| *Note.* Means and standard deviations of perceived threat at pre-training and post-training toward the disliked person as a function of condition (compassion, reappraisal, Italian). SD = Standard Deviation | | | | | | |

**No impact of interventions on prosocial behaviors measured by the Dictator Game**

To test norm-based prosocial behavior, we used the Dictator Game. A 2*3 ANCOVA with a between-subjects factor condition (compassion, reappraisal, Italian), a within-subjects factor target person (disliked person, neutral person), and two covariates related to the training practice (audio recording practice, informal practice) was conducted. Results indicated that there was a main effect of the person, *F*(1,103) = 21.17, *p* <.001, *η_p_*^2^ = .17; participants gave generally more to the neutral person than the disliked person. In line with previous findings (Engel, 2011), overall participants gave 43.7% of their endowment to the neutral person whereas 22.7% was given to the disliked person (see full details in Supplementary Table 4). No main effect of condition was found, *F*(2,103) = 0.64, *p* = .53, *η_p_*^2^ = .01. In addition, neither the interaction condition × target person, *F*(2,103) = 1.58, *p* = .21, *η_p_*^2^ = .03, nor the follow up comparisons between conditions regarding the prosocial behaviors toward the disliked person were significant (all *p*_s_ ≥ .46). Similarly, post-hoc Tukey’s HSD comparisons showed no significant difference between conditions for the money given to the neutral person (*p*_s_ ≥ .62). These findings align with previous results showing that compassion training did not increase monetary distribution in norm-based prosocial behavior as measured by the Dictator Game (Leiberg et al., 2011).

| **Supplementary Table 4**  *Dictator Game* | | | | | | | |
| --- | --- | --- | --- | --- | --- | --- | --- |
|  |  | **Compassion** | | **Reappraisal** | | **Italian** | |
|  |  | (n = 37) | | (n = 36) | | (n = 35) | |
|  |  | *Mean* | *SD* | *Mean* | *SD* | *Mean* | *SD* |
| **Disliked person** | | 2.41 | 2.99 | 2.28 | 3.3 | 2.11 | 2.74 |
| **Neutral person** | | 4.16 | 2.30 | 5 | 3.15 | 3.94 | 2.40 |
| *Note.* Means and standard deviations of the money given to the disliked person and to the neutral person during the Dictator Game as a function of condition (compassion, reappraisal, Italian). SD = Standard Deviation | | | | | | | |

**Emotion regulation interventions reduce competitive behavior**

The economic behaviors of the Inequality Game (IG; Klimecki et al., 2016) as well as the feedbacks sent to the other players were analyzed using two repeated-measures 3*2 ANCOVAs including a between-subjects factor condition (compassion, reappraisal, Italian) and a within-subjects factor other player (fair player, unfair player). The covariates in these analyses were the audio recording practice and the informal practice. Five participants did not believe they were interacting with real people during the IG and were excluded. This reduced the sample size for these analyses to 103 individuals. Analyses on economic choices revealed a significant main effect of other player, *F*(1,98) = 14.14, *p* <.001, *η_p_*^2^= .13, with economic behaviors being more cooperative toward the fair player (*M* = 4.78, *SD* = 2.40) than the unfair other (*M* = 2.5, *SD* = 4.08). No main effect of the condition was found, *F*(2,100) = 1.50, *p* = .22, *η_p_*^2^= .03. The interaction condition × other player was not significant, *F*(2,100) = 1.33, *p* = .27, *η_p_*^2^= .03. The effects of the covariates were not significant, *p*_s_ ≥ .21. A 3*2 repeated-measures ANCOVA on feedbacks also revealed a main effect of other player, *F*(1,98) = 5.44, *p* = .02, *η_p_*^2^= .05 : participants sent nicer feedbacks (and less derogatory feedbacks) to the fair player (*M* = 4.74, *SD* = 3.96) compared to the unfair player (*M* = 2.89, *SD* = 4.88). No main effect of the conditions, *F*(2,98) = 0.64, *p* = .52, *η_p_*^2^= .01, nor an interaction condition × other player were found, *F*(2,98) = 1.83, *p* = .17, *η_p_*^2^= .04. The covariates audio recording practice and the informal practice were significant: *F*(1,98) = 4.88, *p* = .03, *η_p_*^2^= .05, and *F*(1,98) = 4.64, *p* = .03, *η_p_*^2^= .05, respectively. Due to the significance of the covariates, we conducted subsequently Spearman correlations to observe whether audio recording practice and the informal practice were associated to feedbacks in the Inequality Game. The correlations analyses indicated that there was no significant association between the training practice and feedbacks sent in the IG, *p*_s_ ≥ .11. In addition, none of the planned contrasts calculated for the economic behaviors or for the feedbacks when comparing conditions were significant, (all *t*_s_(98) ≤ 0.11*,* all *p*_s_ ≥ .11).

Based on their economic choices toward the fair and the unfair players, participants were categorized according to their preferences for cooperative, sanctioning, or competitive economic behavior (see Inequality Game section in the methods). This categorization included only 88 participants as 15 participants did not show any systematic preference (ambiguous). The majority of participants (n = 67) were categorized as cooperative while 18 were sanctioning, and 3 competitive (see Supplementary Figure 1).

**Supplementary Figure 1**

*Preferences for Economic Behavior in the Inequality Game*


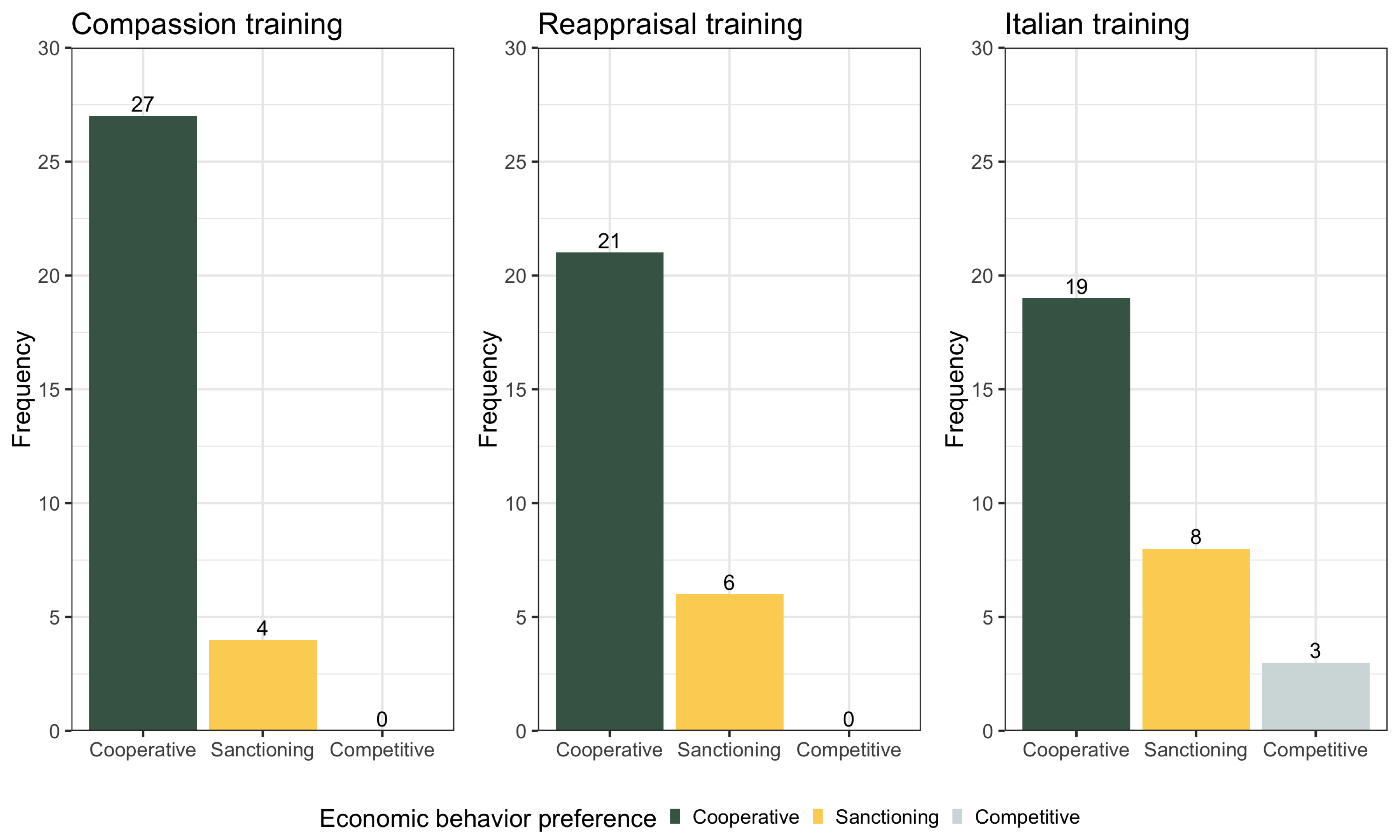


*Note*. Classification of participants based on their preferences in economic behavior as a function of condition (compassion, reappraisal, Italian).

To test whether the distribution of behaviors was modified by the experimental condition, a chi-square test was calculated. It revealed a trend for a relationship between the conditions (compassion, reappraisal, Italian training) and participants’ preferences for economic behaviors (cooperative, sanctioning, competitive), *χ^2^*(4) = 8.42, *p* = .077. Pearson’s chi-squared tests were then executed for each condition (compassion, reappraisal, Italian training) to determine whether participants’ preferences for economic behaviors (cooperative, sanctioning, competitive) differ from behavior predicted by chance distribution. These analyses indicated that more participants than expected by chance preferred to be cooperative toward both players. In line with previous results (Klimecki et al., 2016, 2018) this significant effect was found among compassion trainees, reappraisal trainees, as well as Italian trainees, (all *p*_s_ < .001). Conversely, the frequency of sanctioning behaviors of participants was found not to deviate from sanctioning behaviors predicted by chance distribution among compassion trainees, reappraisal trainees, as well as Italian trainees, (all *p*_s_ ≥ .40). Finally, regarding competitive behaviors, participants in the emotion regulation interventions (compassion training, and reappraisal training) showed significantly less competitive behaviors than expected by chance distribution (all *p*_s_ <.05), whereas participants in the Italian condition did not differ from the chance distribution, *p* = .22.

In addition to economic behaviors and feedbacks measures, IG also provided an assessment of characteristics (reliability, fairness, and pleasantness) that participants attributed to the other players. Besides, participants also evaluated to what extent they were able to take the other player’s perspective (perspective-taking). Means and standard deviations are presented in Supplementary Table 5. Concerning the evaluation of the unfair player, three repeated-measures ANCOVA were executed for each of the characteristics assessed (fairness, pleasantness, and reliability). The three analyses included a between-subjects factor condition (compassion, reappraisal, Italian training), a within-subjects factor fairness (fair, unfair player), and two covariates (audio recording practice, informal practice). Results revealed that across the three analyses, the factor fairness was significant (all *p*_s_ <.001), with the fair player being evaluated more positively (fairer, more pleasant, more reliable) than the unfair player see Supplementary Table 5 for means and standard deviations. In the three ANCOVA, no significance was found for the factor condition (*p*_s_ ≥. 70), the interaction condition × fairness (all *p*_s_ ≥ .42), nor the covariates, (all *p*_s_ ≥ .17).

In order to explore whether there were differences between conditions, post hoc analyses (Tukey HSD) were calculated: none of the comparison were significant across the three characteristics reported for the fair player and the unfair player (all *p*_s_ ≥ .50).

To test whether participants differed in their self-reported degree of perspective taking regarding both other players, a 2*3 repeated-measures ANCOVA with a between-subjects factor condition (compassion, reappraisal, Italian training), with the within-subjects factor fairness (fair, unfair player) and the two covariates related to training practice (audio recording practice, informal practice) was conducted. A main effect for the fairness factor was found, *F*(1, 98) = 11.88, *p* <.001, *η_p_*^2^ = .11, participants were more able to adopt the fair player’s perspective (*M* = 7.13, *SD* = 2.68) than the perspective of the unfair player (*M* = 5.10, *SD* = 3.31). Results also indicated no significant effect of condition, *F*(2,98) = 1.29, *p* = .28, *η_p_*^2^ = .03, no significant interaction condition × fairness, *F*(2,98) = 0.79, *p* = .46, *η_p_*^2^ = .02, and no significant effect of the covariates, *p*_s_ ≥ .79. Moreover, computed contrasts revealed no difference between conditions regarding their ability to take the

perspective of the other player (fair and unfair player), *p*_s_ ≥ .11

| **Supplementary Table 5**  *Characteristics Attributed to Players in the Inequality Game* | | | | | | | | |
| --- | --- | --- | --- | --- | --- | --- | --- | --- |
|  |  | **Compassion** | | | **Reappraisal** | | **Italian** | |
|  |  | (n = 35) | | | (n = 34) | | (n = 34) | |
|  |  | *Mean* | | *SD* | *Mean* | *SD* | *Mean* | *SD* |
| **Fair** | | |  |  |  |  |  |  |
|  | Reliability | | 7.13 | 2.38 | 6.77 | 2.85 | 6.71 | 3.14 |
|  | Fairness | | 6.71 | 2.62 | 6.97 | 2.39 | 6.92 | 3.08 |
|  | Pleasantness | | 7.26 | 2.47 | 7.06 | 2.62 | 6.91 | 2.98 |
|  | Perspective-Taking | | 7.45 | 2.43 | 7.06 | 2.66 | 6.86 | 2.98 |
| **Unfair** | | |  |  |  |  |  |  |
|  | Reliability | | 3.93 | 3.21 | 3.53 | 3.22 | 4.32 | 3.54 |
|  | Fairness | | 4.15 | 3.05 | 3.55 | 2.94 | 3.34 | 2.89 |
|  | Pleasantness | | 3.62 | 3.07 | 3.18 | 2.73 | 3.28 | 2.77 |
|  | Perspective-Taking | | 5.59 | 3.23 | 5.52 | 3.14 | 4.18 | 3.48 |
| *Note*. Means and standard deviations of characteristics attributed to the fair player and the unfair player as a function of condition (compassion, reappraisal, Italian). SD = Standard Deviation | | | | | | | | |
